# Novel personalized pathway-based metabolomics models reveal key metabolic pathways for breast cancer diagnosis

**DOI:** 10.1186/s13073-016-0289-9

**Published:** 2016-03-31

**Authors:** Sijia Huang, Nicole Chong, Nathan E. Lewis, Wei Jia, Guoxiang Xie, Lana X. Garmire

**Affiliations:** Molecular Biosciences and Bioengineering Graduate Program, University of Hawaii at Manoa, Honolulu, HI 96822 USA; Epidemiology Program, University of Hawaii Cancer Center, Honolulu, HI 96813 USA; Department of Microbiology, University of Hawaii at Manoa, Honolulu, HI 96822 USA; Department of Pediatrics, University of California, San Diego, CA 92093 USA; Novo Nordisk Foundation Center for Biosustainability at the University of California, San Diego School of Medicine, San Diego, CA 92093 USA

## Abstract

**Background:**

More accurate diagnostic methods are pressingly needed to diagnose breast cancer, the most common malignant cancer in women worldwide. Blood-based metabolomics is a promising diagnostic method for breast cancer. However, many metabolic biomarkers are difficult to replicate among studies.

**Methods:**

We propose that higher-order functional representation of metabolomics data, such as pathway-based metabolomic features, can be used as robust biomarkers for breast cancer. Towards this, we have developed a new computational method that uses personalized pathway dysregulation scores for disease diagnosis. We applied this method to predict breast cancer occurrence, in combination with correlation feature selection (CFS) and classification methods.

**Results:**

The resulting all-stage and early-stage diagnosis models are highly accurate in two sets of testing blood samples, with average AUCs (Area Under the Curve, a receiver operating characteristic curve) of 0.968 and 0.934, sensitivities of 0.946 and 0.954, and specificities of 0.934 and 0.918. These two metabolomics-based pathway models are further validated by RNA-Seq-based TCGA (The Cancer Genome Atlas) breast cancer data, with AUCs of 0.995 and 0.993. Moreover, important metabolic pathways, such as taurine and hypotaurine metabolism and the alanine, aspartate, and glutamate pathway, are revealed as critical biological pathways for early diagnosis of breast cancer.

**Conclusions:**

We have successfully developed a new type of pathway-based model to study metabolomics data for disease diagnosis. Applying this method to blood-based breast cancer metabolomics data, we have discovered crucial metabolic pathway signatures for breast cancer diagnosis, especially early diagnosis. Further, this modeling approach may be generalized to other omics data types for disease diagnosis.

**Electronic supplementary material:**

The online version of this article (doi:10.1186/s13073-016-0289-9) contains supplementary material, which is available to authorized users.

## Background

Breast cancer is the most frequently diagnosed cancer in women worldwide excluding skin cancer and it is ranked second for deaths among cancer patients [1]. Early diagnosis of breast cancer is crucial for patient prognosis. Currently, however, clinically diagnosed breast tumors have a median size of 2 to 2.5 cm [2], which are likely to be later stage (stage III) breast tumors that have already metastasized to axillary lymph nodes. A highly accurate diagnostic test for breast cancer is currently lacking. The standard mammography test has sensitivities of merely 54 to 77 % [3]. Other diagnostic tools such as ultrasound, computed tomography (CT), and magnetic resonance imaging (MRI) are slightly more sensitive but are costly. There is a pressing need for more accurate, cost-efficient, and non-invasive alternative methods for breast cancer diagnosis.

Meeting these criteria, metabolomics has quickly risen as a new method in the cancer biomarker field. As the final products of various biological processes, metabolites hold promise as accurate biomarkers that reflect upstream biological events such as genetic mutations and environmental changes [4]. Discovery of altered metabolites and pathways will help us to gain better understanding of dysregulated metabolism in tumor initiation and progression. Previous metabolomics studies have shown that certain metabolites can successfully differentiate patients from normal controls or even classify sub-populations of certain diseases, including breast cancer [5–13]. For example, glutamate was found enriched in breast cancer patients and the glutamate-to-glutamine ratio was significantly correlated with estrogen receptor status [12]. Serum profiles of breast cancer patients showed that histidine, glucose, and lipids were strongly correlated with breast cancer relapse with a predictive accuracy of 75 % [13]. However, similar to other types of biomarkers, metabolomics biomarker results are difficult to replicate among different studies for a combination of reasons, such as the heterogeneity of the populations and study sizes, variability of the experimental protocols, noise in the metabolomics data, as well as biological variations in the turnover rates of metabolites.

Given the observation that metabolites and enzymes involved in the same biological processes are often dysregulated together in cancer [14], we hypothesize that higher-order quantitative representations of metabolomics features, such as pathway-based metabolomics features, are coherent surrogates of metabolomics biomarkers that provide more information on biological functions. To our knowledge, this idea had not been implemented in the context of metabolomics data, although it had been proposed before in other types of omics data analysis, such as transcriptomics and genetics (genome-wide association studies and exome-sequencing) data. Towards this, we have developed a completely personalized, novel computational method for pathway-based metabolomics data analysis using the non-parametric principle curve approach [15]. We integrate metabolite features as pathway features and subject them to feature selection and machine-learning classifications. This methodology is applied to identify breast cancer diagnosis biomarkers, especially for early pathological stages. The resulting classification models are highly accurate for breast cancer all-stage diagnosis (area under the curve (AUC) = 0.986) and early-stage diagnosis (AUC = 0.995) in the plasma training set. Moreover, these models predict equally impressively in plasma testing and serum validation samples, with AUCs of 0.923 and 0.995, respectively, for the all-stage diagnosis and 0.905 and 0.902, respectively, for early-stage diagnosis. We have discovered several pathways critical for the early diagnosis of breast cancer, including taurine and hypotaurine metabolism and alanine, aspartate, and glutamate metabolism.

## Methods

### Study population

Three data sets are used in this study: two metabolomics data sets from our own group and one RNA-Seq data set from The Cancer genome Atlas (TCGA) breast cancers. The first metabolomics cohort is composed of 132 breast cancer and 76 control plasma samples and the second independent set comprises 103 breast cancer and 31 control serum samples. All samples were obtained from City of Hope Hospital (COH). This study was approved by the institutional review boards of the City of Hope National Medical Center. All participants signed an informed consent before they participated in the study. Additionally, we downloaded TCGA breast cancer RNA-Seq data from 1082 tumor and 98 tumour adjacent normal controls [16] from the TCGA data portal (https://tcga-data.nci.nih.gov/tcga/). Patient characteristics, staging of disease, and other parameters are shown in Table 1.

**Table 1:** Summary of patient and clinical characteristics

	COH plasma				COH serum		TCGA paired RNA-Seq	
	Training set		Test set		Test set		Test set	
Characteristics	Breast cancer	Healthy control	Breast cancer	Healthy control	Breast cancer	Healthy control	Breast cancer	Healthy control
Number of samples	106	61	26	15	103	31	98	98
Age (years; median, range)	53, 31–73	34, 21–40	54.5, 36–72	37, 21–40	52, 32–72	36, 18–49	56, 30–90	56, 30–90
Stage (number of patients)								
I	16		3		18		16	
II	40		9		49		60	
III	38		8		54		21	
IV	11		6		19		1	
Race (number of patients)								
Asian	14		3		14		1	1
Black	5	12	4	1	6	5	6	6
White	76	28	18	9	69	21	90	90
Latino		21		5		5		
Native					1			
Other	10		1		13		1	1

### Data set configurations for diagnostic model training, validation, and testing

For the all-stage diagnosis model, we used 80 % of the plasma (106) and 80 % of the control (61) samples as the training data. We employed three testing data sets, including: (1) the remaining 20 % of the plasma (26) and 20 % of the control (15) samples as the first hold-out testing data; (2) the entire 103 breast cancer and 31 control serum samples; (3) a cohort of 98 pairs of age-matched breast cancer TCGA RNA-Seq data. There is no sample overlap between the training and test sets. To train the early stage diagnosis model, we used the stage I (15 samples) and II (37 samples) subsets of the training data in the all-stage diagnosis model described above, in combination with the 61 healthy control samples.

### Collection and storage of blood serum and plasma

Fasting serum and plasma specimens were collected in the morning before breakfast from all the participants. The samples from controls were obtained from healthy volunteers. The breast cancer patients were newly diagnosed and were not recurrent or on any medication prior to sample collection. All samples were placed into clean tubes and immediately stored within 2 h of collection at −80 °C until analysis.

### Metabolic profiling

Liquid chromatography/time-of-flight mass spectrometry (LC-TOFMS) and gas chromatography/time-of-flight mass spectrometry (GC-TOFMS) were used for the metabolomics profiling of all blood samples in the study. The profiling procedure included sample preparation, metabolite separation and detection, metabolomics data pre-processing, metabolite annotation, and, finally, statistical analysis for biomarker identification. To eliminate batch effects, all of the plasma samples were processed in one batch, as were all of the serum samples. All annotated metabolites from GC-TOFMS and LC-TOFMS data sets were combined and exported to SIMCA-P+ 12.0 software (Umetrics, Umeå, Sweden) for multivariate statistical analysis.

### Pathway mapping of metabolites

The names of metabolites are standardized by linking them to Human Metabolome Database (HMDB) IDs, with consideration of synonyms. A comprehensive master file was created which contains the mapping information between 310 human metabolic pathways and affiliated metabolites. Pathway and metabolite information were extracted from HMDB [17], Small Molecule Pathway Database (SMPDB) [18], Kyoto Encyclopedia of Genes and Genomes (KEGG) [19], Recon 2 [20], IPA (QIAGEN’s Ingenuity® Pathway Analysis, IPA®, QIAGEN, Redwood City, http://www.ingenuity.com/), FLink (Frequency weighted Links, http://ncbi.nlm.nih.gov/Structure/flink/flink.cgi) and PubChem [21]. Most of the metabolites could be mapped to pathways by the master file. The remaining unmapped metabolites were manually searched for in the literature.

### Pathifier algorithm

We used the R package *pathifier* [22] to perform pathway-based metabolite sets analysis. Details about *pathifier* are described elsewhere [22, 23]. Briefly, this algorithm transfers information from the metabolite level to pathway level by inferring the pathway dysregulation score (PDS) for each sample in each pathway. This PDS is an individualized pathway-level measurement of abnormality. The normal condition samples are utilized to construct a principal curve, which is then smoothed. Every sample is projected onto the smoothed principal curve and the PDS is the normalized projection distance for each pathway of each sample. If the sample deviates further from others in a particular pathway, then the projection distance to the curve is larger and leads to a higher PDS for this pathway.

### Feature selection and evaluation of classification models

For feature selection from the training data, we used the correlation feature selection (CFS) method implemented in Weka [24] with tenfold cross-validation. CFS is a machine-learning method that selects features with the highest correlation to responses and lowest correlation with other selected features [25]. In the tenfold cross-validation step, training data were split into ten parts, nine of which were used as the actual training set while the remaining part was used as the validation set, such that a set of features were selected by CFS. We repeated this process ten times among different parts and kept the features that were selected ten out of ten times (100 %). To select the best-suited classifier, we evaluated the performance of three classification methods (logistic regression, support vector machine (SVM), and random forest) on the training data set for the same set of CFS-selected features. We used a comprehensive list of metrics that include AUC, sensitivity, specificity, Matthew’s correlation coefficient (MCC), and F1-statistic.

### TCGA RNA-Seq analysis

Breast cancer TCGA RNA-Seq data were downloaded from the data portal (https://tcga-data.nci.nih.gov/tcga/) on 23 October 2015 [16]. We included 1082 breast cancer samples with 98 control samples. For pathway level analysis, we implemented the *pathifier* algorithm on the RNA-Seq data and applied limma’s differential *t*-test to compare the pathway level results with our study. For metabolite level analysis, the enzyme (gene) information for featured metabolites was extracted from KEGG and SMPDB. Limma’s differential *t*-tests were used for calculation of the *p* values for each enzyme (gene). Barplots were used for comparison between metabolites and the related enzyme (gene) in breast cancer and normal samples.

### Metabolite-based model comparison

We built the metabolite-based model on the same plasma training data set. We conducted feature selection and classification the same way as for pathway-based models so that the results are comparable. Specifically, we used the CFS method implemented in Weka with a tenfold cross-validation for feature selection. We implemented logistic regression models for all-stage and early-stage classification to compare with pathway-based models.

### Power analysis of the diagnosis model

To ensure the adequacy of our pathway-based metabolomics model, we calculated the sample size and statistical power using the module implemented in MetaboAnalyst [26], where the implementation was described by van Iterson et al. [27].

### Data availability

All the input metabolomics data used for this study have been deposited in Metabolomics Workbench (http://metabolomicsworkbench.org/; project ID PR000284). Additionally, the metabolites mapped to pathways are included in Additional file 1. The R scripts for pathway mapping, PDS matrix generation, and logistic regression are available at http://www2.hawaii.edu/~lgarmire/MetaboloPathwayModel.html.

## Results

### Data sets and the analysis workflow

Three data sets are used in this study: two of them are our own metabolomics profiling data sets from independent plasma and serum samples and the third is the TCGA breast cancer RNA-Seq data set (to test the generalization of the pathway-based model across data types). The metabolomics data include newly diagnosed pre-treatment samples comprising (1) 132 breast cancer and 76 control plasma samples and (2) 103 breast cancer and 31 control serum samples. For the two plasma and serum sample data sets, we conducted metabolomics experiments by both liquid chromatography time-of-flight mass spectrometry (LC-TOFMS) and gas chromatography time-of-flight mass spectrometry (GC-TOFMS). According to the power analysis tool in MetaboAnalyst [26], the study achieves a power of 0.84 (Additional file 2: Figure S1), supporting the adequacy of the metabolomics data. Physiological and clinical information, such as age, ethnicity, and tumor stage for the plasma, serum data, and TCGA sets are summarized in Table 1.

**Fig. 1:**
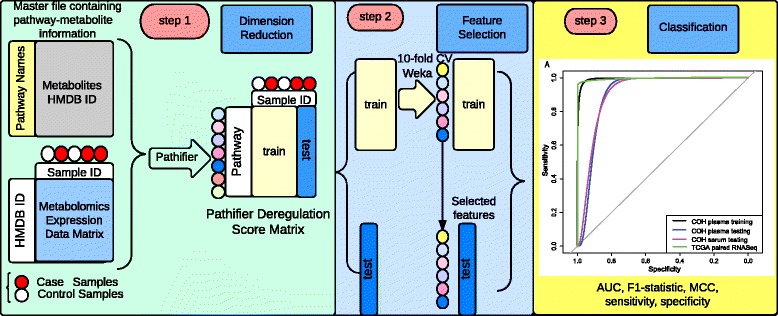
The workflow of the pathway-based metabolomics data analysis. Step 1: conversion from metabolite- to pathway-based metabolomics data. The input data include the master file containing pathway-metabolite mapping information, the metabolomics profiling data, and the normal/tumor classification vector. The metabolomics-level data are transformed to pathway-level data by the *pathifier* algorithm. The output file of *pathifier* is the pathway dysregulation score matrix, within which each score measures the deregulation of a specific pathway for a specific sample. Step 2: model construction. Qualified COH plasma samples are split by 80/20 for training and holdout testing data. Correlation feature selection (CFS) is used for feature selection and the logistic regression model is used for classification. Tenfold cross-validation (*10-fold CV*) is applied with CFS feature selection in the plasma training data set. Two models are constructed: an all-stage diagnostic model and an early-stage diagnostic model. Step 3: model evaluation. The model performance is assessed using receiver operating characteristic (ROC) curves and various metrics, including AUC, MCC, sensitivity, specificity, and F1-statistic

To analyze the metabolomics data, we have developed a novel computational pipeline that identifies pathway-based biomarkers for blood-based breast cancer diagnosis (Fig. 1). The essence of the approach is to transform metabolite-level information to completely personalized pathway-level information. The overall workflow of the pathway-based model and the analysis process is as follows.

First, metabolites are mapped to their standardized Human Metabolome Database (HMDB) IDs and the pathway–metabolite relationships are summarized in a master file from multiple resources, including HMDB, Kyoto Encyclopedia of Genes and Genomes (KEGG), Small Molecule Pathway Database (SMPDB), IPA, FLink, Recon 2, and PubChem. Next, we used the *pathifier* algorithm to convert the raw metabolite-based data matrix to the pathway-based matrix that contains pathway dysregulation scores (PDS). Pathifier is a non-parametric method for dimension reduction, where a one-dimensional principle curve is derived from a cloud of data points in the high-dimensional space. The PDS is a metric for the degree of pathway abnormality per patient and it is the distance on the principle curve from the starting point to the point projected by a particular and individualized pathway [15, 22]. A PDS ranges from 0 to 1, where a score closer to 1 indicates a more aberrant pathway. Then, we used the PDS matrix from 80 % of the qualified plasma set to train classification models. We selected the plasma set to train the classification models as it has a larger sample size and more complete information of tumor stages. The details of feature selection and classification to train the models and model testing with three different data sets are described in the following sections.

### Metabolic pathway-based all-stage diagnostic model for breast cancer

**Fig. 2:**
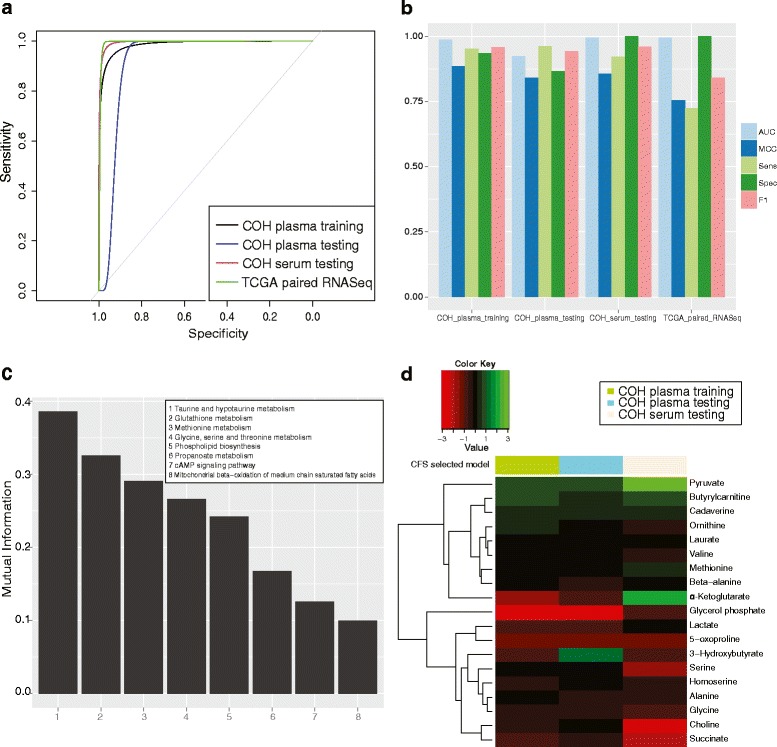
The performance of the all-stage diagnosis model for breast cancer. We used 80 % of the controls and cases in the COH plasma data set to train the model. The remaining COH plasma data (20 %) and the COH serum data set were used as the test set and validation set. **a** Receiver operating characteristic (ROC) curves for the all-stage breast cancer diagnosis from different data sets. **b** AUC, MCC, sensitivity, specificity, and F1-statistic to measure the performance of the all-stage diagnosis model. **c** Mutual information for pathway features selected by the all-stage diagnosis model. **d**. Log fold change of metabolites associated with the selected pathway features determined by comparing cases and controls across different data sets

We first investigated the metabolomics-based pathways as biomarkers to predict breast cancers composed of all stages of tumors (Fig. 2). To select the best set of features that are maximally relevant and minimally redundant, we used CFS with tenfold cross-validation on the plasma training data set, which is composed of 80 % of the breast cancer and 80 % of the healthy control samples. With these selected features (Fig. 2c), we evaluated three widely used classification methods (logistic regression, SVM, and random forest) on the plasma training data set. The resulting performance metric AUC (0.986) shows that logistic regression performs the best among the three methods (Additional file 3: Table S1). We thus used the logistic model as the model of choice to evaluate three other testing data sets: the 20 % hold-out plasma testing samples, the entire serum sample set, and a cohort of 98 pairs of age-matched breast cancer RNA-Seq data from TCGA. Note for TCGA data that we generated the PDS and extracted the values for the same features as the training data set. Although these three data sets are generated from different populations and technology platforms, our hypothesis is that pathway-based features should represent true biology and the model based on metabolomics data should, therefore, be generally predictive.

The resulting metabolic pathway-based diagnostic model performs very well on all three testing data sets, with AUCs of 0.923, 0.995, and 0.9946 in the hold-out plasma testing samples, serum samples, and TCGA RNA-Seq set, respectively (Fig. 2a). Moreover, other statistical metrics, such as the sensitivity, specificity, MCC, and F1-statistic, are also outstanding, confirming the robustness and generality of the pathway-based model (Fig. 2b). The superior performance of the model on the serum metabolomics and TCGA RNA-Seq data sets is surprising. This may be due to the more complete lists of metabolites in the serum data set and genes in the RNA-Seq data set compared with the plasma samples. The good AUC obtained from the age-matched TCGA RNA-Seq data suggests that age is unlikely to be a driving factor leading to the accuracy of the classification from the metabolomics-based pathway-model. Nevertheless, we further examined if age is a dominant confounding factor in the metabolomics training data. For this, we divided the plasma data into two subsets: subset 1 with 35 pairs of age-comparable samples and subset 2 with 97 breast cancer and 41 age-incomparable controls. If diagnosis signals were driven by age, then a model trained on age-incomparable subset 2 would have very poor prediction on subset 1, where the ages among these samples are comparable. However, a new model on age-incomparable subset 2 still achieves a very high AUC of 0.913 on age-comparable subset 1. Thus, the pathway features (Fig. 2c) in the earlier model are predictive of breast cancer diagnosis.

These eight pathway features are listed in the following in descending order with regard to their relevance, as measured by Mutual Information (MI), for diagnosis: taurine and hypotaurine metabolism; glutathione metabolism; methionine metabolism; glycine, serine, and threonine metabolism; phospholipid biosynthesis; propanoate metabolism; cAMP signaling pathway; and mitochondrial beta-oxidation of medium chain saturated fatty acids. Interestingly, none of the pathways has an MI greater than 0.5, indicating the complexity of the disease and the significance of pathways collectively. Among them, taurine and hypotaurine metabolism stands out as the most important pathway (MI = 0.386). Hypotaurine is a product of the enzyme cysteamine dioxygenase, which is involved in protecting against oxidative stress and cancer-induced membrane damage [28, 29]. The taurine and hypotaurine metabolic pathway has been shown to be relevant to multiple types of cancers, such as ovarian, lung, colon, and renal cancers [30–33]. Here, for the first time, we have discovered that taurine and hypotaurine metabolism is also dysregulated in the blood samples of breast cancer. In order to confirm the significance of each pathway at the transcriptome level, we crosschecked pathway-level expression results using TCGA RNA-Seq data. The pathway level results of two data types are consistent overall, as expected (Additional file 3: Table S2). For example, the taurine and hypotaurine metabolism pathway has a significant *p* value of 1.01E-25 for the differential test in the metabolomics data and it is also a top-ranked pathway with a *p* value of 7.40E-9 in the RNA-Seq data.

Next, we identified the measurable metabolites in these selected pathways from both plasma and serum samples and measured their average log fold changes in tumor versus control samples (Fig. 2d; Additional file 3: Table S3a). Hypotaurine is the primary metabolite in the leading significant taurine and hypotaurine pathway, and it is increased by 2.41-fold (0.0086 vs. 0.0025) in the tumor sample compared with the normal plasma sample. Pyruvate, the most central metabolite in the cell and a common component of glycine, serine, and threonine metabolism and taurine and hypotaurine metabolism pathways, is consistently present at higher levels in breast cancer blood samples (Fig. 2d; Additional file 3: Table S3a): it is increased by 1.82-fold in the plasma sample and 2.89-fold in the serum sample compared with control (Fig. 2d; Additional file 3: Table S3a). Interestingly, several amino acids are present at lower levels in cancer samples compared with controls, including succinate (1.69-fold decrease in plasma, 4.58-fold decrease in serum), choline (1.23-fold decrease in plasma, 4.58-fold decrease in serum), serine (2.72-fold decrease in plasma, 1.13-fold decrease in serum), glycine (1.25-fold decrease in plasma, 1.83-fold decrease in serum) and alanine (1.11-fold decrease in plasma, 1.62-fold in serum) (Additional file 3: Table S3a). Decreased levels of glycine and alanine in plasma and serum of breast cancer patients have been reported before [34, 35]. Choline, serine, and glycine are the major components of glycine, serine, and threonine metabolism, glutathione metabolism, and methionine metabolism, whereas succinate is the major component of propanoate metabolism and the cAMP signaling pathway. Similarly, levels of glycerol-3-phosphate in phospholipid biosynthesis are significantly lower in the cancer samples, with a sixfold decrease in plasma. The comparisons between some key metabolites in our metabolomics study and the corresponding enzymes from TCGA RNA-Seq data are shown in Additional file 2: Figure S2. Overall, the directions of change in metabolite levels are consistent with those of corresponding enzymes.

### Metabolic pathway-based early-stage diagnostic model for breast cancer

**Fig. 3:**
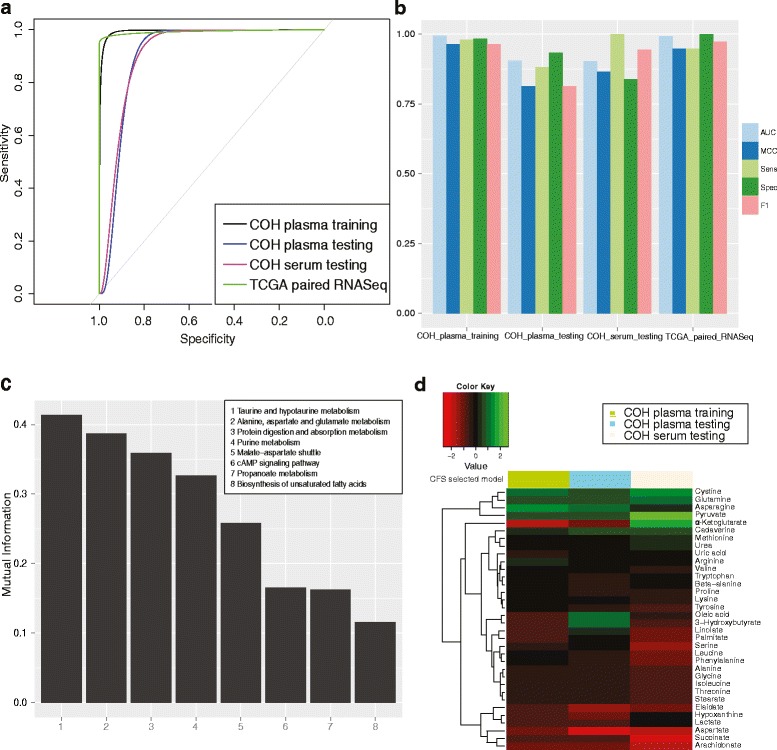
The performance of the early-stage diagnosis model for breast cancer. We used 80 % of the controls and early-stage (stage I and II) cases in the COH plasma data set to train the model. The remaining controls and early stage cases in the COH plasma data set, as well as controls and early stage cases in the COH serum data set, were used as the testing and validation set. **a** Receiver operating characteristic (ROC) curves for the early-stage breast cancer diagnosis from different data sets. **b** AUC, MCC, sensitivity, specificity, and F1-statistic to measure the performance of the early-stage diagnosis model. **c** Mutual information for pathway features selected by the all-stage diagnosis model. **d** Log fold change of metabolites associated with the selected pathway features determined by comparing cases and controls across different data sets

Early detection of breast cancer is critical to improve survival. Due to the small sample size (n = 16) of stage I tumors, we combined the samples in stages I and II as early-stage cancers and constructed a sub-model to diagnose early-stage breast cancer, similar to the previous all-stage diagnosis model. As expected, the pathway-based early-stage diagnostic model performs very well on the training data set, with an AUC of 0.995. Moreover, it also predicts very well on the three testing data sets, with AUCs of 0.905, 0.902, and 0.999 in the 20 % hold-out plasma testing, serum, and TCGA breast cancer samples (Fig. 3a). Other model performance metrics also yield satisfactory results in both data sets, supporting the excellence of the early diagnostic model (Fig. 3b).

Eight key pathways are identified as diagnostic features for early-stage breast cancer detection (Fig. 3a), namely taurine and hypotaurine metabolism, alanine, aspartate, and glutamate metabolism, protein digestion and absorption, purine metabolism, malate-aspartate shuttle, cAMP signaling pathway, propanoate metabolism, and biosynthesis of unsaturated fatty acids (listed in descending order of significance). Similar to the all-stage diagnosis model, taurine and hypotaurine metabolism is again the top-ranked pathway (MI = 0.414; Fig. 3c), indicating its significance as a new signature for early-stage breast cancer detection. Alanine, aspartate, and glutamate metabolism is a new pathway feature selected by the early-stage diagnosis model, largely due to the increase of the intensity of aspartate from 0.063 to 0.182 and decrease of the intensity of asparagine from 0.091 to 0.038 in the cancer and control plasma samples, respectively. This implies a transformational relationship from aspartate to asparagine in cancer. The cAMP signaling pathway has been intrinsically linked to a variety of pathways, such as the PI3K pathway, and antibodies directed against the soluble adenylyl cyclase that catalyzes cAMP production have been shown to be highly specific markers for melanoma [36, 37]. To further confirm the significance of our finding, we calculated the differences in the above eight feature pathways between tumor and control samples using the metabolomics data and TCGA RNA-Seq data. The pathway-level results are significant for both metabolomics and RNA-Seq data sets (Additional file 3: Table S2).

At the metabolite level, some key metabolites are preserved in the early-stage diagnosis sub-model (Fig. 3d) compared with the all-stage model (Fig. 2d). These include cysteine, glutamine, and asparagine, which are present at higher concentrations in early-stage tumor samples, as well as alanine and aspartate, which are decreased during early tumorigenesis. The finding that aspartate, the precursor of beta-alanine [38], is significantly and robustly lower even in early-stage breast cancers is very interesting and further confirms that dysregulations of amino acid metabolism and metabolites are early events associated with breast cancer tumorigenesis [35]. We summarize the average expression of the key metabolites and the differential test *p* values in Additional file 3: Table S3b. We also compare the relationship between the expression of key metabolites from our study and the expression of genes encoding the enzymes that transform those metabolites from the TCGA RNA-Seq data in Additional file 2: Figure S3. Both sets of results show consistent trends in general.

### Integrative analysis of key pathways and metabolites

**Fig. 4:**
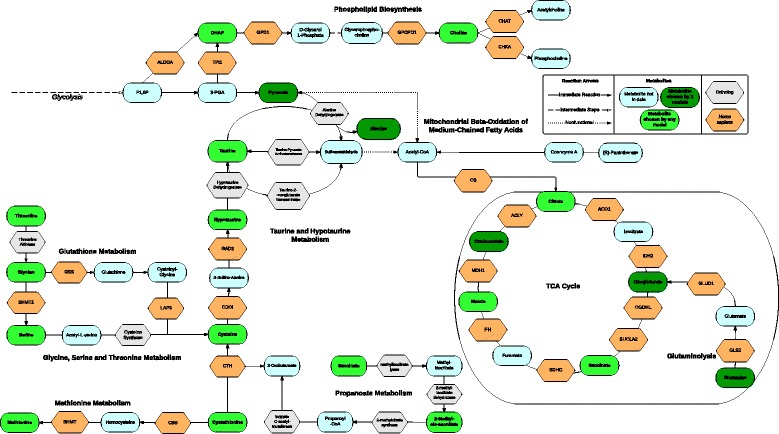
Integrative analysis of pathway features and the associated metabolites. The key pathways and their intersections crucial for breast cancer diagnosis. Metabolites and enzymes are represented with nodes of different shapes and colors, and their relationships are represented by edges

Metabolic regulation is elaborately linked to cancer initiation and progression as proliferating cells demand nutrients for energy production as well as synthesis of genetic materials, proteins, and lipids [4, 14]. Although the feature pathways identified by the diagnostic and early diagnostic models are different, they are nevertheless interconnected in the cellular context (Fig. 4). Alanine, glutamine, and aspartate metabolism are interconnected and we observe consistent trends of decreasing alanine, glutamine, and aspartate levels in cancer vs. normal samples. Moreover, amino acid, glucose, and phospholipid metabolism can be interconnected through glutaminolysis, a process that supplies carbon and nitrogen resources to the growing and proliferating cancer cells [39]. We also summarize the overlap between metabolites from the pathways featured in the all-stage diagnosis and early-stage diagnosis models. Common metabolites important to the two models are beta-alanine, glycine, serine, lactate, succinate, oxoglutarate, alanine, 3-hydroxybutyrate, methionine, valine, cadaverine, and pyruvate, all functionally linked to glutaminolysis (Additional file 2: Figure S4).

### Comparison of pathway-based and metabolite-based metabolomics models

**Fig. 5:**
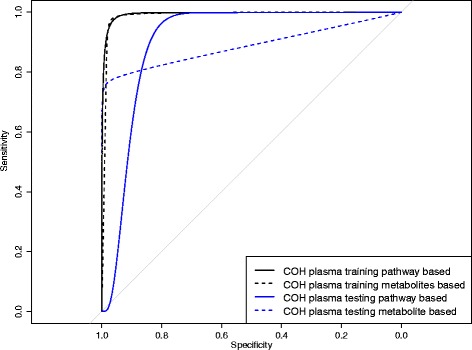
Receiver operating characteristic (ROC) curves comparison of pathway-based model and metabolites-based model among data sets. The same 80 % of early stage (stage I and II) cases and controls from the COH plasma data set used in the early-stage diagnosis model were used for the plasma training set. The remaining 20 % of early stage (stage I and II) cases and controls represent the test set. The metabolite-based model is based on the same tenfold cross-validation CFS selection used for the plasma training set. ROC curves for training and test sets are compared between the plasma-based model and the metabolite-based model among data sets

To evaluate the pathway-based metabolomics diagnosis modeling approach compared with the commonly used metabolite-based approach, we constructed a “baseline” metabolite-based model using exactly the same CFS feature selection and logistic regression steps used in our pathway-based method. Since the AUC values indicate that the early-stage model is less likely to have over-fitting, we used the early-stage breast cancer data to compare the pathway-based and metabolite-based diagnosis models. In the training data set, the pathway-based approach performs slighter better, with an AUC of 0.995 compared with 0.988 in the metabolite-based approach (Fig. 5). A similar trend also exists in the testing data set, where the pathway-based model yields an AUC of 0.905 and the metabolite-based model has an AUC of 0.888 (Fig. 5).

US Food and Drug Administration approval of biomarkers requires the demonstration of the biomarker candidate functions [40]. We thus built single-variate logistic models to show the diagnostic potential of the individual pathway or metabolite features selected by the models. Comparatively, the top pathway features show better disease association than the top metabolite features (Additional file 3: Table S4). In the pathway-based model, taurine and hypotaurine metabolism is the most statistically significant (*p* < 2E-16, *t*-test) followed by the protein digestion and absorption pathway (*p* = 3.5E-10, *t*-test). On the other hand, in the metabolite-based model, the most significant metabolite, cysteine (HMDB00192), has a significant *p* value of 2.22E-9. These results indicate that the top individual pathway feature may have better diagnostic performance than metabolites.

To investigate the effect of the number of pathways on the performance of the pathway-based model, we conducted sensitivity analysis as exemplified by the early-stage diagnosis model. We randomly selected half (51) of the initial 101 pathways within exactly the same training sample sets and applied the same CFS feature selection criteria with tenfold cross-validation. CFS selects six pathways for the early-stage model (Additional file 3: Table S5). We imposed logistic regressions on these selected features and compared the changes in AUCs due to changes in pathways. Reducing the initial number of pathways decreases the performance of the models, as expected. In the training data, the half-size pathway-based early stage diagnosis model has a slight decrease of AUC from 0.995 to 0.948. Such a decrease is more pronounced in the serum testing data, from 0.903 to 0.753. Similar trends are observed for the all-stage diagnosis model.

## Discussion

### Summary of discoveries

Metabolomics provides the most direct measurement of phenotypic changes because it reflects the final molecular result of the combination of all upstream genetic, transcriptomic, and proteomic changes [41]. The relative incomplete coverage of metabolomic measurements has been a challenge for their use as diagnostic classifiers. In this study, we address this challenge by using a new metric of personalized pathway dysregulation score (PDS). This score can interpret the metabolomics data in the context of the metabolic pathways on the individual patient level, thereby enabling us to discriminate the differences in specific pathways between cancer and normal samples. This approach accurately predicted all-stage breast cancer patients from normal controls (AUC = 0.968). It even detected early-stage (stage I and II) breast cancers with excellent accuracy (averaged AUC = 0.904 in two test sets). In addition to the increased power achieved by integrating concerted metabolic changes as described in this paper, our pathway-based classifiers can potentially offer deeper biological insights into which cellular processes are dysregulated in breast cancer. We have discovered novel critical pathways, such as taurine and hypotaurine metabolism and alanine, aspartate, and glutamate metabolism related to glutaminolysis, for the early diagnosis of breast cancer.

### A new paradigm to use pathways as features of biomarker classification models

Conventionally, almost all metabolomics studies aim to identify metabolites as biomarkers. Even among the few studies that involve the systematic pathway approach [42–45], none have developed a computational methodology to employ pathways as input features for the downstream statistical or machine learning modeling of biomarker diagnosis or prognosis.

The disadvantage of using metabolites as predictors of biomarker diagnosis or prognosis models are obvious: low reproducibility. This could be due to various reasons, such as the heterogeneity of the populations and small study sizes, variability of the experimental protocols, and technical noise in the metabolomics data. In fact, we compared the multiple studies that had attempted to identify metabolites in blood as biomarkers for breast cancer previously [34, 46–48] and found little overlap or even controversies among them (Additional file 2: Figure S5) [13, 34, 35, 47–52]. On the contrary, many metabolites in the featured pathways that we have found with our method coincide with previous reports, such as increases in alanine, pyruvate, and lactate as well as decreases in choline in cancer samples. Thus, the pathway-based method is more tolerant of heterogeneity in populations compared with the metabolite-based biomarker approach. The tolerance of the pathway-based method for population heterogeneity is also manifested through the pathway features being able to accommodate age differences. The models predict fairly well on three different sets of test data, even when the ages are matched. Moreover, the biologically motivated feature selection approach offers systems level and biological level insights, which the metabolite-based models lack. Such system-level knowledge is very critical as we move forward towards developing intervention strategies for cancer prevention or therapeutic strategies for cancer treatment. Biological systems are highly robust with redundant components and attacking the higher-level structures such as pathways offers a better strategy than changing the expression of lower-level components such as genes or metabolites.

The workflow that we propose here is a fully personalized pathway-based diagnostic modeling framework for metabolomics data. Moreover, it is compatible with the conventional metabolite-based predictive modeling approach after the step of input matrix transformation. This methodology represents generalization of the pathway-based predictive modeling philosophy, which we had exemplified earlier using transcriptomics and clinical data to predict breast cancer prognosis [23]. The most distinguished characteristic of our method is that it summarizes the contribution of potentially correlated metabolites in the same pathway into a single metric, the PDS, on a patient by patient basis. Our method not only preserves the individual patient information before classification but also gives a direct numerical value (rather than the rank) per pathway per patient. Doing so provides great flexibility for using pathways as features for various downstream analyses, exemplified here as diagnosis biomarker modeling. The applications go far beyond disease diagnosis though. For example, one could also use the new data matrix of PDSs to perform clustering or survival analysis. On the other hand, other bioinformatics tools for metabolomics analysis, such as MetaboAnalyst [26] and Metabolite Set Enrichment Analysis [53], either use pathway enrichment post hoc or lose individual patients’ values during the set enrichment analysis.

Perhaps the most powerful feature of this modeling approach is that the pathway features may be generalized to other omics platforms despite the differences in experimental protocols, what is measured (metabolites, mRNAs, proteins, etc.), and their units. Here we have demonstrated that the pathway features obtained from metabolomics data have excellent predictive performance in TCGA breast cancer RNA-Seq data, where both the sample sources and technical platform are different from the metabolomics data sets. Moreover, by projecting metabolite profiles onto pathway profiles, metabolomics data can be integrated with other types of omics data, such as RNA-Seq gene expression, DNA methylation, and copy number variation data.

### Important discoveries of altered pathways during carcinogenesis

Our results demonstrate that taurine and hypotaurine metabolism is the most indicative pathway for breast cancer diagnosis. Taurine, converted from hypotaurine by hypotaurine dehydrogenase, is intricately linked with alanine and glutamate metabolism (Fig. 4). Although this is the first report of the possible importance of this significant pathway in breast cancer early diagnosis, many lines of evidence suggest this is a critical pathway in tumor development. Hypotaurine is known to modify the indices of oxidative stress and membrane damage, both of which are associated with cancer [28, 54]. Additionally, others have linked this pathway to worse prognosis in ovarian, kidney, colon, and lung adenocarcinoma [30, 32, 33, 55]. Moreover, glutamate decarboxylase 1, a key enzyme in taurine and hypotaurine metabolism, has been identified as a tissue biomarker for benign and malignant prostate cancer [56].

We also found alanine, aspartate, and glutamate metabolism together with the malate-aspartate shuttle to be significant pathways in the early-stage diagnosis model. Aspartate is the key metabolite that shows significantly lowered levels in breast cancer blood samples (Additional file 3: Table S3b). It is produced from oxaloacetate by a transamination process. and participates in the urea cycle to facilitate the removal of ammonia as well as acting in the biosynthesis of pyrimidine for translocation of NADH into mitochondria. Interestingly, the lower level of aspartate in the blood is conversely associated with increased aspartate in breast cancer tissues and cell lines [57], suggesting that the aspartate pool in the blood is utilized to supply more aspartate in breast cancer cells. Consistent with this hypothesis, asparagine synthetase, the enzyme that generates asparagine from aspartate, was overexpressed under glucose deprivation in pancreatic cancer cells to protect against apoptosis [58].

### Perspectives and future work

In this study, we have proposed a new and personalized pathway-based approach to integrate metabolite-level metabolomics data in the diagnosis of breast cancer. The success of this type of pathway model first relies on data obtained through a profiling (rather than targeted) approach where as many metabolites/genes as possible are recorded. Compared with other omics data types, metabolomics data are much less standardized across different studies and data repositories are lacking [59, 60]. A community effort needs to be made to improve data sharing in order to accumulate statistically well-powered data sets to predict disease diagnosis and prognosis. To drive our modeling approach towards clinical diagnosis, we are planning to build a large database to store the metabolomics profiles as references. In the model construction step, samples will be labeled as cancer/normal classes are used and their individual pathway scores (normalized scores between 0 and 1) will be calculated as inputs subject to feature selection and classification step. When a new sample arrives, the metabolite profile will be normalized relative to the database and a new vector of PDSs will be calculated after the same metabolite-to-pathway transformation. The classification model can then call for the probability of this new sample being normal or cancerous. Depending on the accuracy of prediction in the new sample, we can elect to incorporate it into the training data set and re-train the model, thus improving the predictive power of the model over time. Moreover, from the new patient’s PDS profile we can also infer the aberrant pathways and identify problematic metabolites (and associated enzymes) for this specific patient. Therefore, the discoveries could be used for not only diagnosis prediction but also precision medicine.

## Conclusions

We have successfully developed a new type of pathway-based model that uses metabolomics data for disease diagnosis. Applying this method to blood-based breast cancer metabolomics data, we were able to discover crucial metabolic pathway signatures for breast cancer diagnosis, which may be valuable for diagnostic tests and therapeutic interventions [61, 62]. Further, this modeling approach can be broadly applicable to other omics data types for disease diagnosis.

## Additional files

Additional file 1: Mapped metabolites names. (CSV 5 kb) Additional file 2: Figure S1. Power analysis and sample size estimation plot. **Figure S2.** Bar plot comparing the key metabolites in the all-stage diagnosis model to the expressions of corresponding enzymes in TCGA breast cancer RNA-Seq data. The enzymes (genes) for these metabolites were extracted from KEGG and SMPDB. *P* values were calculated using differential tests in *Limma*. ****P* < 0.001. **Figure S3.** Bar plot comparing the key metabolites in the early-stage prediction model with the expression levels of corresponding enzymes in TCGA breast cancer RNA-Seq data. The enzymes (genes) for these metabolites were extracted from KEGG and SMPDB. *P* values were calculated using differential tests in *Limma*. ****P* < 0.001. **Figure S4.** Venn diagram of the metabolites from the selected pathways in two models (all-stage diagnosis and early-stage diagnosis). **Figure S5.** Metabolites detected as biomarkers for breast cancers by different studies. Study1 (serum), Jobard et al. [46]. Study2 (serum), de Leoz et al. [49]. Study3 (serum), Oakman et al. [48]. Study4 (serum), Asiago et al. [50]. Study5 (serum), Tenori et al. [13]. Study6 (plasma), Miyagi et al. [35]. Study7 (cell line), Yang et al. [51]. Study8 (plasma), Shen et al. [34]. Study9 (plasma), Miller et al. [52]. Study10 (serum), Poschke et al. [47]. (PDF 1375 kb) Additional file 3: Table S1. Comparison of logistic regression, SVM and random forest performance in the plasma training data set. **Table S2.** Pathway significance and relative log fold changes in our metabolomics data and TCGA breast cancer RNA-Seq data. **Table S3.** Detected metabolites and their differential test results among the two models. **a** All-stage diagnosis model. **b** Early-stage diagnosis model. **Table S4.** Single-variate logistic analysis of metabolites or pathways selected as features in the metabolite-based or pathway-based early-stage diagnosis model. **Table S5.** Comparison of pathway features in the full-size (101 input pathways) and half-size (51 input pathways) pathway-based early-stage diagnosis models. (DOCX 34 kb)

## Abbreviations

AUC: area under the curve
CFS: correlation feature selection
COH: City of Hope Hospital
GC-TOFMS: gas chromatography/time-of-flight mass spectrometry
HMDB: Human Metabolome Database
KEGG: Kyoto Encyclopedia of Genes and Genomes
LC-TOFMS: liquid chromatography/time-of-flight mass spectrometry
MCC: Matthew’s correlation coefficient
PDS: pathway deregulation score
SMPDB: Small Molecule Pathway Database
SVM: support vector machine
TCGA: The Cancer Genome Atlas
