# Supplementary material for: Novel personalized pathway-based metabolomics models reveal key metabolic pathways for breast cancer diagnosis
Source: Genome Med. 2016 Mar 31;8:34. doi: 10.1186/s13073-016-0289-9 (PMC4818393; doi:10.1186/s13073-016-0289-9)

Supplementary Figure 1

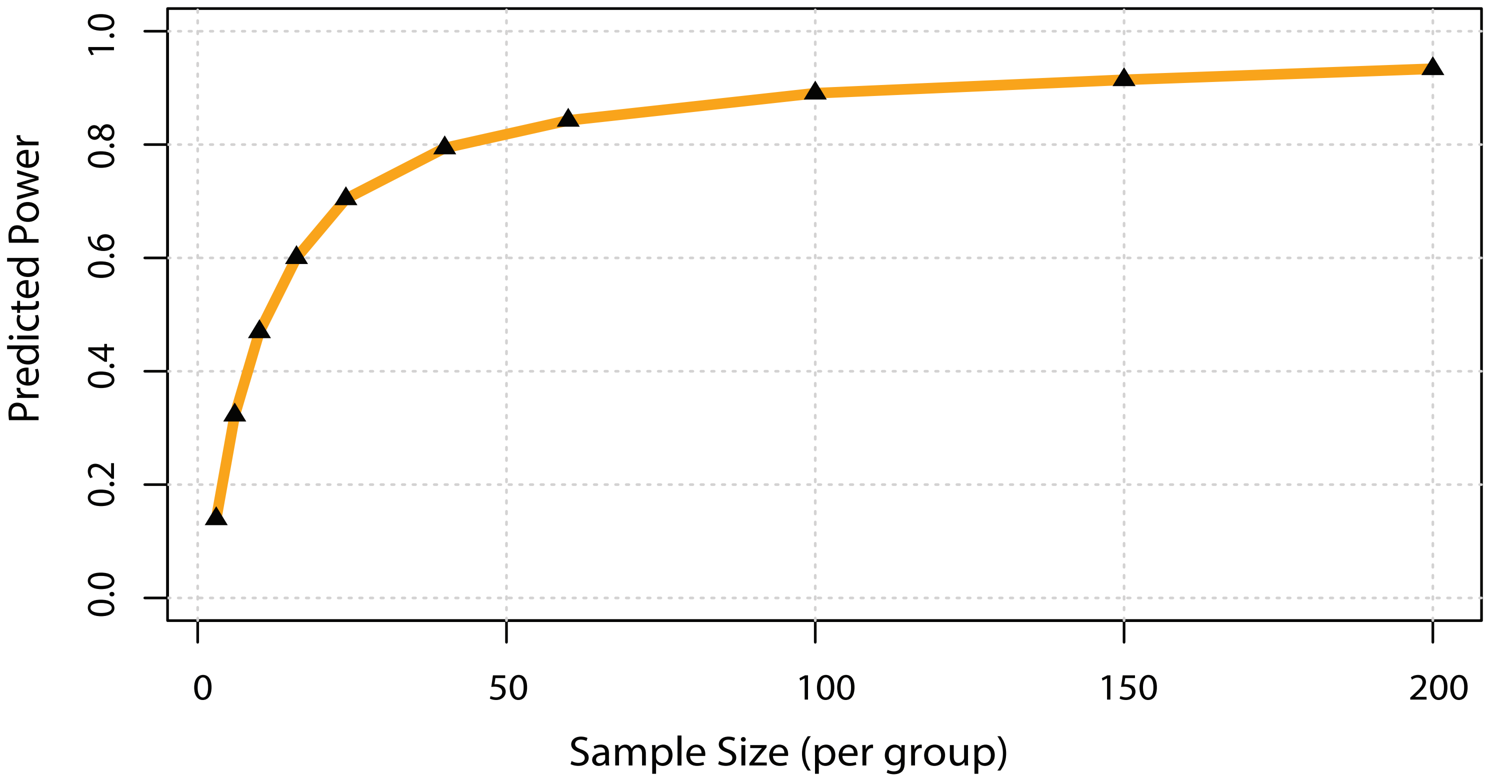

Supplementary Figure 2

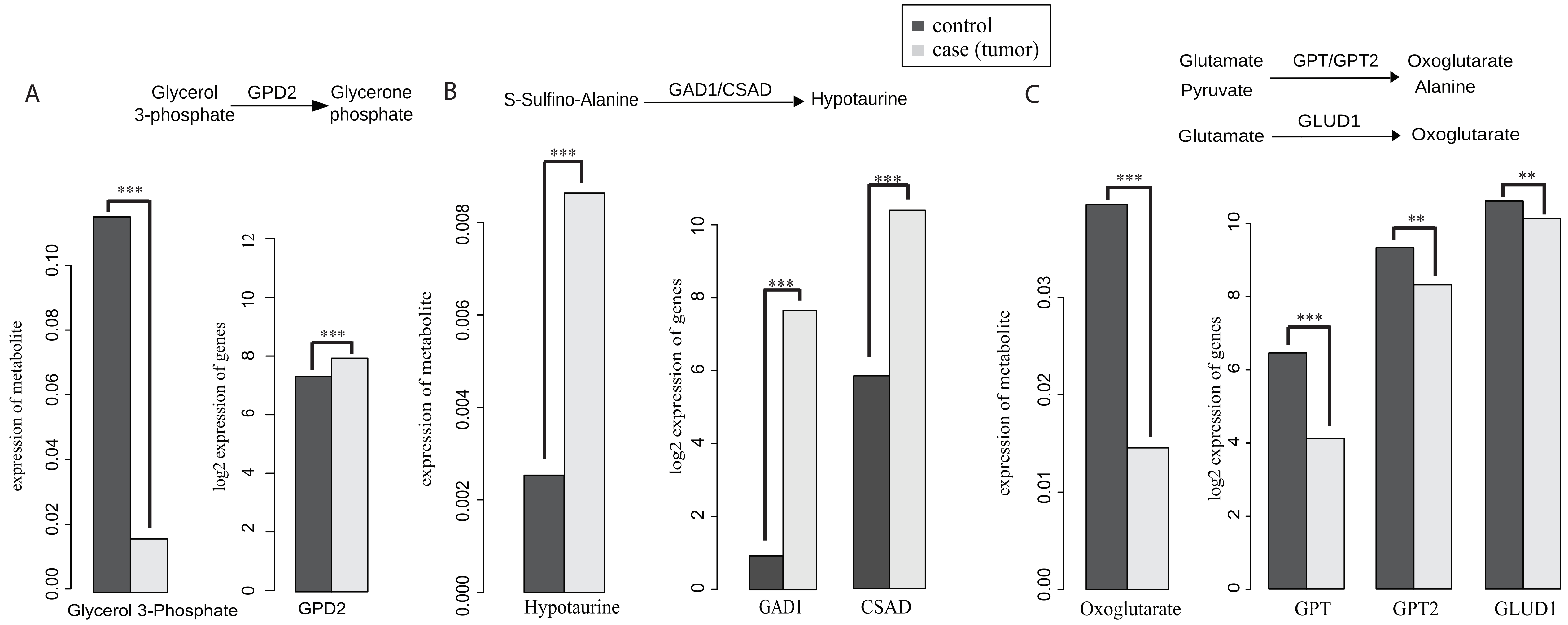

Supplementary Figure 3

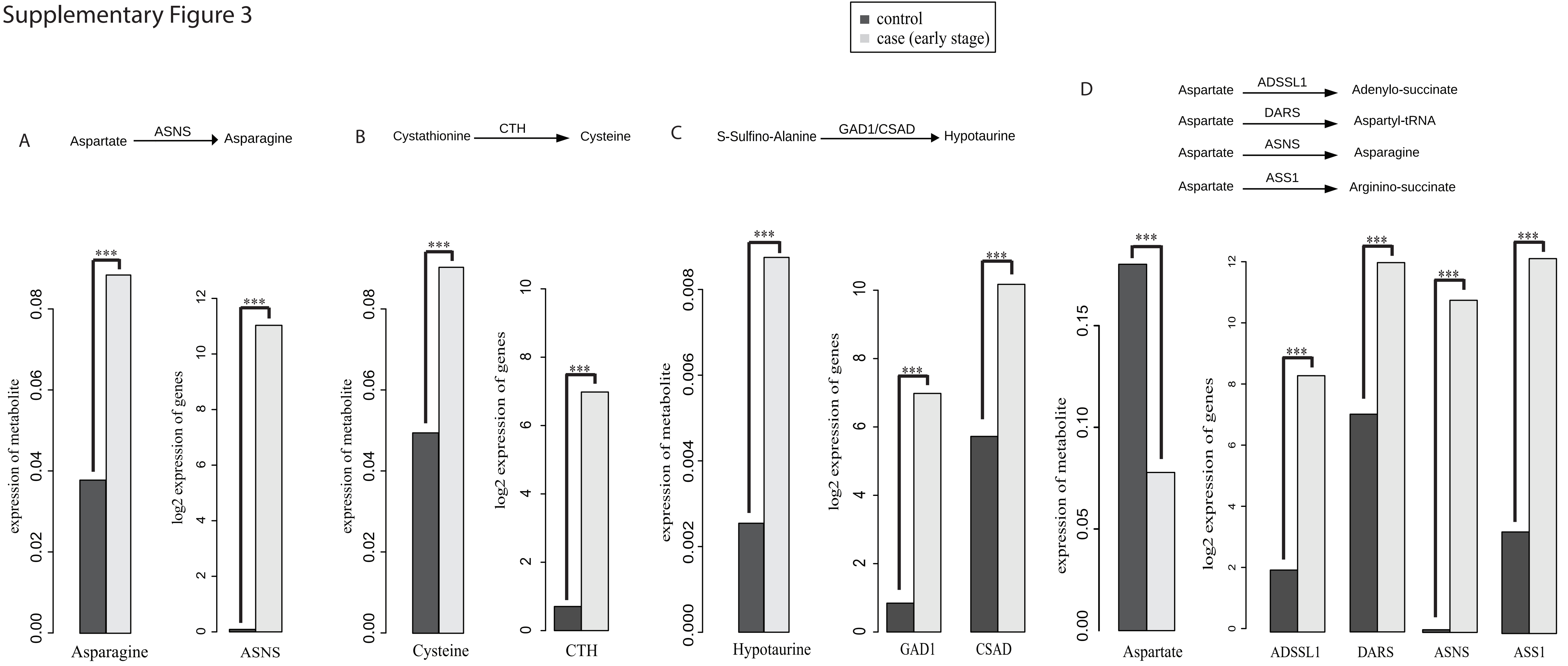

Supplementary Figure 4

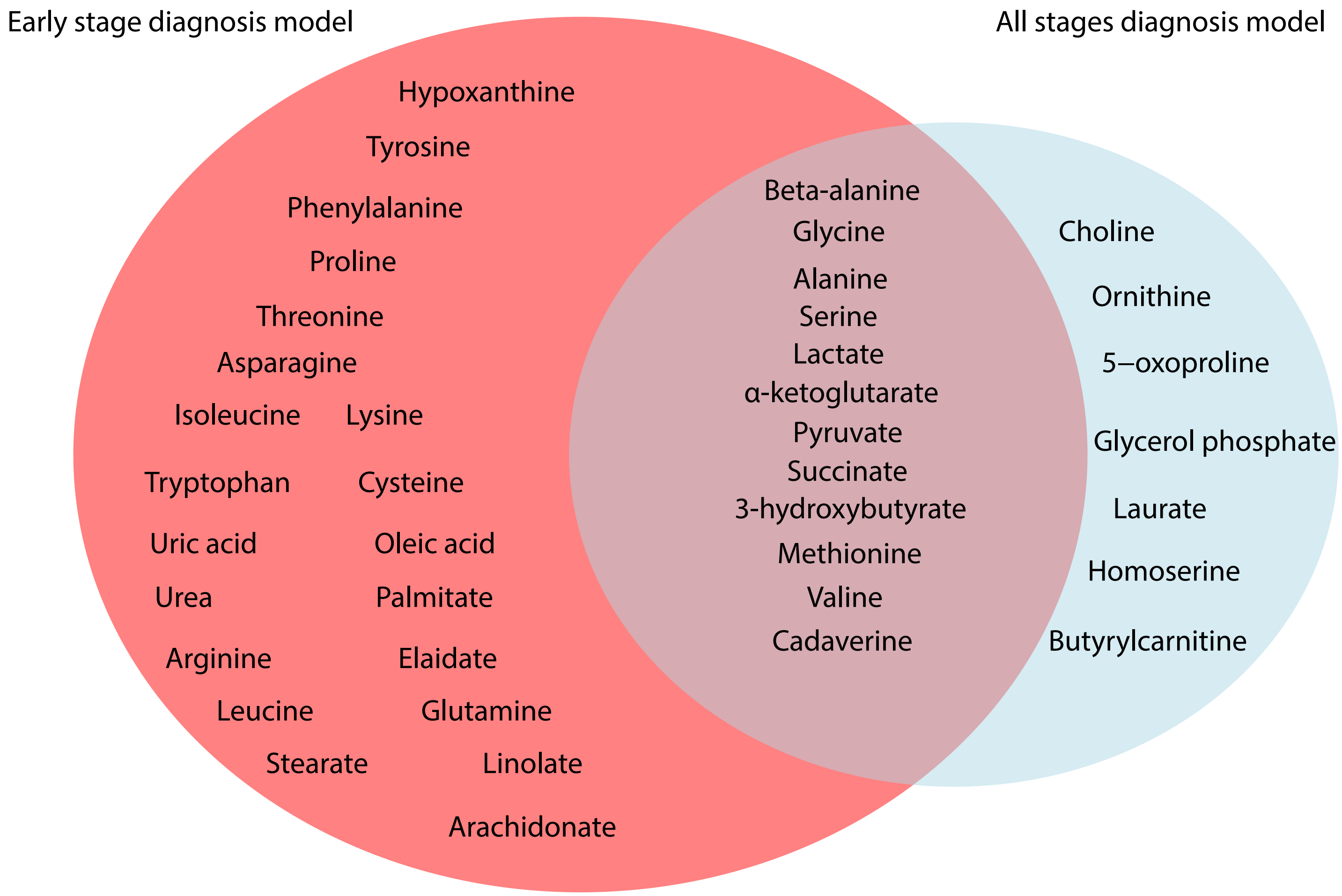

Supplementary Figure 5

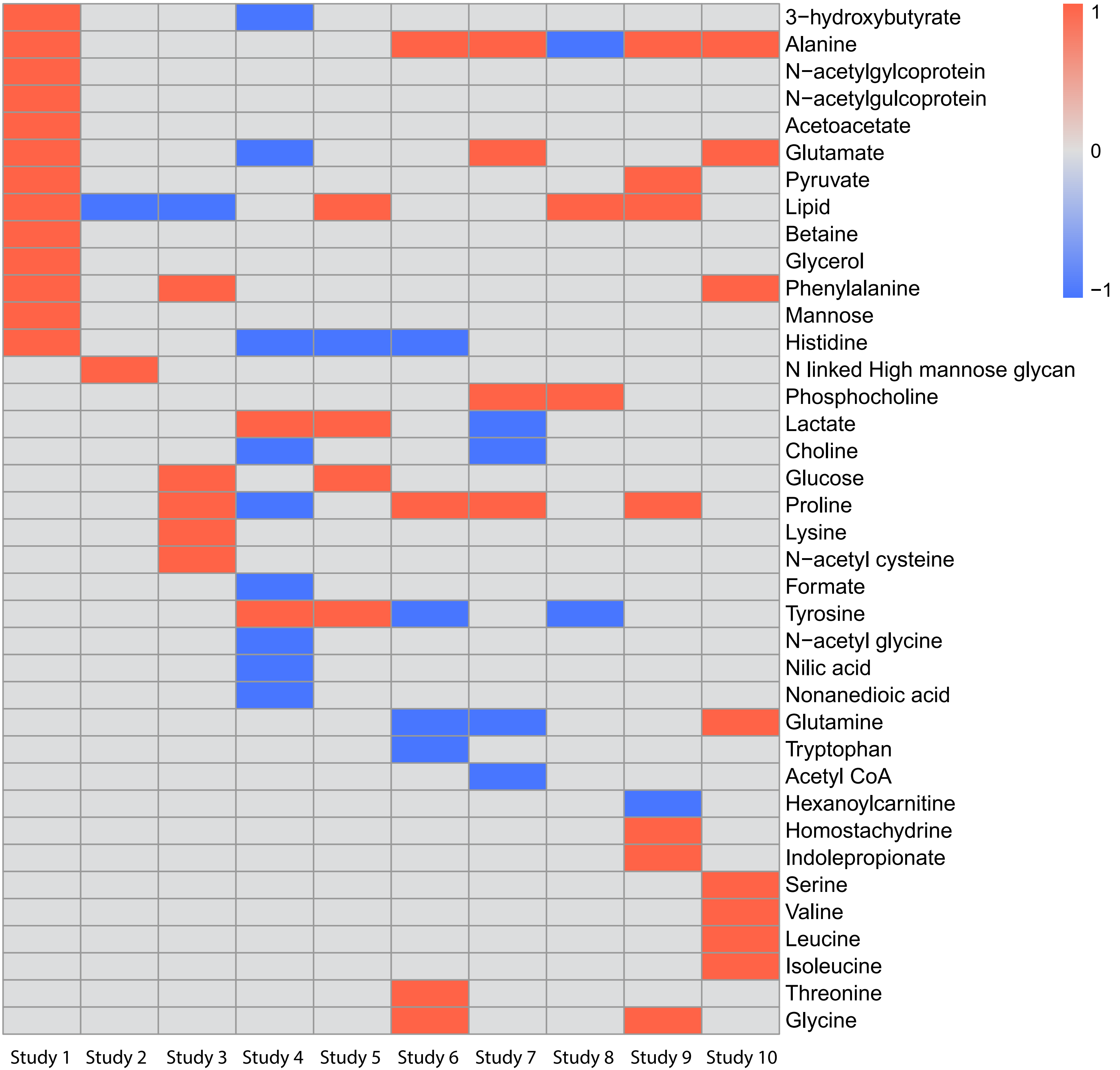

Supplement: Additional file 2: Figure S1. — Power analysis and sample size estimation plot. Figure S2. Bar plot comparing the key metabolites in the all-stage diagnosis model to the expressions of corresponding enzymes in TCGA breast cancer RNA-Seq data. The enzymes (genes) for these metabolites were extracted from KEGG and SMPDB. P values were calculated using differential tests in Limma. ***P < 0.001. Figure S3. Bar plot comparing the key metabolites in the early-stage prediction model with the expression levels of corresponding enzymes in TCGA breast cancer RNA-Seq data. The enzymes (genes) for these metabolites were extracted from KEGG and SMPDB. P values were calculated using differential tests in Limma. ***P < 0.001. Figure S4. Venn diagram of the metabolites from the selected pathways in two models (all-stage diagnosis and early-stage diagnosis). Figure S5. Metabolites detected as biomarkers for breast cancers by different studies. Study1 (serum), Jobard et al. [46]. Study2 (serum), de Leoz et al. [49]. Study3 (serum), Oakman et al. [48]. Study4 (serum), Asiago et al. [50]. Study5 (serum), Tenori et al. [13]. Study6 (plasma), Miyagi et al. [35]. Study7 (cell line), Yang et al. [51]. Study8 (plasma), Shen et al. [34]. Study9 (plasma), Miller et al. [52]. Study10 (serum), Poschke et al. [47]. (PDF 1375 kb) [file 13073_2016_289_MOESM2_ESM.pdf]
