# Supplementary material for: Novel personalized pathway-based metabolomics models reveal key metabolic pathways for breast cancer diagnosis
Source: Genome Med. 2016 Mar 31;8:34. doi: 10.1186/s13073-016-0289-9 (PMC4818393; doi:10.1186/s13073-016-0289-9)
Supplement: Additional file 3: Table S1. — Comparison of logistic regression, SVM and random forest performance in the plasma training data set. Table S2. Pathway significance and relative log fold changes in our metabolomics data and TCGA breast cancer RNA-Seq data. Table S3. Detected metabolites and their differential test results among the two models. a All-stage diagnosis model. b Early-stage diagnosis model. Table S4. Single-variate logistic analysis of metabolites or pathways selected as features in the metabolite-based or pathway-based early-stage diagnosis model. Table S5. Comparison of pathway features in the full-size (101 input pathways) and half-size (51 input pathways) pathway-based early-stage diagnosis models. (DOCX 34 kb) [file 13073_2016_289_MOESM3_ESM.docx]

**Supplementary Tables**

**Supplementary Table I: Comparison of logistic regression, SVM and Random Forest performance in plasma training data set**

**Supplementary Table II: Pathway significances and relative log fold changes in our metabolomics data and TCGA breast cancer RNA-Seq data**

**Supplementary Table III: Detected metabolites and their differential test results among the two models**

A: all-stage diagnosis model

B: early-stage diagnosis model

**Supplementary Table IV: Single-variate logistic analysis of metabolites or pathways selected as features in the metabolite-based or pathway-based early-stage diagnosis model.**

**Supplementary Table V: Comparison of pathway features in the full-size (101 input pathways) and half-size (51 input pathways) pathway-based early-stage diagnosis models**

# Supplementary Tables

**Supplementary Table I:** AUC comparison of different model building approaches in plasma training dataset

Diagnosis model

| AUC (data set/ model) | Logistic | SVM | Random Forest |
| --- | --- | --- | --- |
| Plasma training | 0.9857 | 0.9726 | 0.9851 |

Early stage diagnosis model

| AUC (data set/ model) | Logistic | SVM | Random Forest |
| --- | --- | --- | --- |
| Plasma training | 0.9946 | 0.982 | 0.9826 |

**Supplementary Table II:** Pathway significances and relative log fold changes in our metabolomics data and TCGA breast cancer RNA-Seq data.

| Pathway | RNASeq logFC | Metabolomics logFC | RNASeq adj.pval | Metabolomics adj.pval |
| --- | --- | --- | --- | --- |
| Diagnosis |  |  |  |  |
| Taurine and Hypotaurine metabolism | 0.12 | 1.42 | 7.40E-09 | 1.01E-25 |
| Glutathione metabolism | 0.20 | 1.33 | 3.49E-07 | 1.73E-22 |
| Methionine metabolism | 0.20 | 1.12 | 3.63E-12 | 4.41E-16 |
| Glycine, Serine and Threonine metabolism | 0.26 | 0.89 | 2.56E-26 | 2.31E-10 |
| Phospholipid biosynthesis | 0.28 | 0.92 | 9.09E-28 | 3.46E-11 |
| Propanoate metabolism | 0.32 | 0.65 | 4.60E-24 | 5.67E-06 |
| CAMP signaling pathway | 0.27 | 0.62 | 4.31E-31 | 1.50E-05 |
| Mitochondrial beta-oxidation of medium chain saturated fatty acids | 0.18 | 0.64 | 2.63E-10 | 7.92E-06 |
| Early stage prediction |  |  |  |  |
| Taurine and Hypotaurine metabolism | 0.12 | 1.42 | 7.40E-09 | 1.21E-25 |
| Alanine, Aspartate and Glutamate metabolism | 0.14 | 1.07 | 3.16E-10 | 1.26E-14 |
| Purine metabolism | 0.16 | 0.90 | 3.83E-11 | 1.24E-10 |
| Malate-Aspartate shuttle | 0.08 | 0.33 | 4.01E-02 | 2.46E-02 |
| CAMP signaling pathway | 0.27 | 0.62 | 4.31E-31 | 1.50E-05 |
| Propanoate metabolism | 0.32 | 0.65 | 4.60E-24 | 5.67E-06 |
| Biosynthesis of unsaturated fatty acids | 0.12 | 0.41 | 1.54E-05 | 4.01E-03 |

**Supplementary Table III:** Detected metabolites and their differential test results among the two models

A: all-stage diagnosis model

| Metabolites | ID | Adj.pval(BH) | Expr_tumor | | Expr_normal |
| --- | --- | --- | --- | --- | --- |
| Glycerol 3-phosphate | HMDB00126 | 3.78E-31 | | 0.02 | 0.12 |
| Pyroglutamic acid | HMDB00267 | 1.24E-28 | | 1.34 | 3.06 |
| Cysteine | HMDB00574 | 1.04E-22 | | 0.1 | 0.05 |
| Hypotaurine | HMDB00965 | 2.30E-19 | | 0.01 | 0 |
| Lactate | HMDB00190 | 1.34E-09 | | 46.06 | 80.84 |
| Pyruvate | HMDB00243 | 3.55E-09 | | 1.03 | 0.57 |
| Oxoglutarate | HMDB00208 | 3.05E-07 | | 0.01 | 0.04 |
| Succinate | HMDB00254 | 6.45E-06 | | 0.03 | 0.06 |
| Sarcosine | HMDB00271 | 1.21E-04 | | 0.13 | 0.08 |
| Butyrylcarnitine | HMDB02013 | 4.74E-04 | | 0.03 | 0.02 |
| Cadaverine | HMDB02322 | 7.33E-03 | | 0.02 | 0.01 |
| Choline | HMDB00097 | 1.02E-02 | | 0.07 | 0.09 |
| Cystathionine | HMDB00099 | 1.60E-02 | | 0.09 | 0.06 |
| Serine | HMDB00187 | 2.59E-02 | | 1.11 | 1.25 |
| Ornithine | HMDB00214 | 3.04E-02 | | 0.78 | 0.65 |
| Glycine | HMDB00123 | 6.19E-02 | | 1.76 | 2.19 |
| Alanine | HMDB00161 | 1.45E-01 | | 11.55 | 12.8 |
| Homoserine | HMDB00719 | 1.58E-01 | | 0.01 | 0.01 |
| 3-Hydroxybutyrate | HMDB00357 | 2.20E-01 | | 1.49 | 2.14 |
| Aminohippurate | HMDB01867 | 2.31E-01 | | 0.03 | 0.03 |
| Glycerylphosphorylethanolamine | HMDB00114 | 2.55E-01 | | 0.02 | 0.02 |
| Hendecanoate | HMDB00947 | 2.63E-01 | | 0.03 | 0.05 |
| alpha-Methylaconitate | HMDB06357 | 2.73E-01 | | 0.01 | 0.01 |
| Dihydroxyacetone phosphate | HMDB01473 | 3.57E-01 | | 0.02 | 0.02 |
| Valine | HMDB00883 | 3.78E-01 | | 0.46 | 0.44 |
| Epinephrine | HMDB00068 | 5.91E-01 | | 0.01 | 0.01 |
| Hexanoylglycine | HMDB00701 | 6.74E-01 | | 0.02 | 0.02 |
| Dodecanoate | HMDB00638 | 7.01E-01 | | 0.08 | 0.08 |
| Beta-Alanine | HMDB00056 | 7.41E-01 | | 0.01 | 0.01 |
| Methionine | HMDB00696 | 7.98E-01 | | 0.22 | 0.23 |

B: early-stage diagnosis model

| Metabolites | ID | Adj.pval(BH) | Expr_tumor | Expr_normal |
| --- | --- | --- | --- | --- |
| Asparagine | HMDB00168 | 1.27E-18 | 0.09 | 0.04 |
| Cysteine | HMDB00574 | 1.92E-17 | 0.1 | 0.05 |
| Hypotaurine | HMDB00965 | 1.92E-17 | 0.01 | 0 |
| Cystine | HMDB00192 | 6.36E-14 | 0.06 | 0.03 |
| Arachidonic acid | HMDB01043 | 2.28E-10 | 0.01 | 0.03 |
| Glutamine | HMDB00641 | 1.01E-09 | 0.35 | 0.21 |
| Lysine | HMDB00182 | 3.67E-09 | 0.98 | 0.94 |
| Lactate | HMDB00190 | 2.95E-06 | 44.28 | 80.84 |
| Glyoxylate | HMDB00119 | 5.30E-06 | 0.05 | 0.03 |
| Pyruvate | HMDB00243 | 5.41E-06 | 0.99 | 0.57 |
| Isoleucine | HMDB00172 | 4.29E-05 | 0.03 | 0.05 |
| Succinate | HMDB00254 | 2.49E-04 | 0.03 | 0.06 |
| Oxoglutarate | HMDB00208 | 2.65E-04 | 0.01 | 0.04 |
| Threonine | HMDB00167 | 4.44E-04 | 0.74 | 0.99 |
| Stearate | HMDB00827 | 2.28E-03 | 0.5 | 0.68 |
| Aspartate | HMDB00191 | 1.62E-02 | 0.08 | 0.18 |
| Palmitate | HMDB00220 | 2.35E-02 | 1.59 | 2.35 |
| Cadaverine | HMDB02322 | 3.52E-02 | 0.02 | 0.01 |
| Alanine | HMDB00161 | 4.48E-02 | 10.95 | 12.8 |
| Arginine | HMDB00517 | 5.00E-02 | 0.06 | 0.05 |
| Elaidate | HMDB00573 | 5.89E-02 | 0.01 | 0.02 |
| Serine | HMDB00187 | 7.52E-02 | 1.1 | 1.25 |
| Phenylalanine | HMDB00159 | 7.63E-02 | 0.65 | 0.73 |
| Hypoxanthine | HMDB00157 | 8.79E-02 | 0.02 | 0.04 |
| Uric acid | HMDB00289 | 1.53E-01 | 0.39 | 0.47 |
| Linoleate | HMDB00673 | 1.61E-01 | 0.04 | 0.05 |
| Glycine | HMDB00123 | 2.97E-01 | 1.84 | 2.19 |
| Oxalacetate | HMDB00223 | 3.71E-01 | 0.11 | 0.11 |
| 3-Hydroxybutyrate | HMDB00357 | 4.39E-01 | 1.53 | 2.14 |
| Urea | HMDB00294 | 4.64E-01 | 22.9 | 21.22 |
| alpha-Methylaconitate | HMDB06357 | 5.15E-01 | 0.01 | 0.01 |
| Oleate | HMDB00207 | 5.72E-01 | 0.13 | 0.16 |
| Proline | HMDB00162 | 5.76E-01 | 6.58 | 6.97 |
| Valine | HMDB00883 | 6.10E-01 | 0.46 | 0.44 |
| 5-Phosphoribosylamine | HMDB01128 | 6.34E-01 | 0.48 | 0.52 |
| Tyrosine | HMDB00158 | 6.91E-01 | 1.19 | 1.23 |
| Methionine | HMDB00696 | 8.65E-01 | 0.22 | 0.23 |
| Epinephrine | HMDB00068 | 9.11E-01 | 0 | 0.01 |
| Leucine | HMDB00687 | 9.28E-01 | 2.87 | 2.91 |
| Tryptophan | HMDB00929 | 9.28E-01 | 0.64 | 0.65 |
| Beta-Alanine | HMDB00056 | 9.79E-01 | 0.01 | 0.01 |
| Guanine | HMDB00132 | 9.79E-01 | 0.02 | 0.02 |
| Ethylmethylacetate | HMDB02176 | 9.79E-01 | 0.01 | 0.01 |

**Supplementary Table IV:** Single-variate logistic analysis of metabolites or pathways selected as features in the metabolite-based or pathway-based early-stage diagnosis model

Metabolite-based model:

| Features | Names | Estimate Coefficient | Std.Error | t value | Pr(>\|t\|) |
| --- | --- | --- | --- | --- | --- |
| HMDB00192 | Cysteine | -1.21E+01 | 1.86E+00 | -6.513 | 2.22E-09 |
| HMDB00126 | Glycerol 3-phosphate | 3.18E+00 | 5.66E-01 | 5.618 | 3.45E-07 |
| HMDB00086 | Glycerophosphocholine | 1.75E+00 | 3.26E-01 | 5.370 | 4.38E-07 |
| HMDB00641 | Glutamine | -1.43E+00 | 2.93E-01 | -4.881 | 3.56E-06 |
| HMDB00191 | Aspartate | 3.38E-01 | 1.96E-01 | 1.724 | 8.75E-02 |

Pathway-based model:

| Features | | Estimate | Std.Error | | t value | Pr(>\|t\|) |
| --- | --- | --- | --- | --- | --- | --- |
| Taurine And Hypotaurine Metabolism | | 3.26E+00 | 2.54E-01 | | 12.80 | <2E-16 |
| Protein Digestion And Absorption | | 2.56E+00 | 3.71E-01 | | 6.892 | 3.51E-10 |
| Alanine Aspartate Glutamate Metabolism | | 2.56E+00 | 5.27E-01 | | 4.858 | 3.92E-06 |
| Purine Metabolism | | 2.13E+00 | 5.36E-01 | | 3.969 | 1.28E-04 |
| Biosynthesis Of Unsaturated Fatty Acids | | 1.75E+00 | 4.65E-01 | | 3.754 | 2.79E-04 |
| CAMP Signaling Pathway | | 2.53E+00 | 7.41E-01 | | 3.418 | 8.82E-04 |
| Propanoate Metabolism | | 8.17E-01 | 2.63E-01 | | 3.112 | 2.37E-03 |
| Malate-Aspartate Shuttle | | 2.02E-01 | 4.77E-01 | | 0.423 | 6.73E-01 |
|  |  | |  |  | |  |

Estimate: Estimate coefficients

Std.Error: Standard error for estimated coefficients

t-value: T-test statistic for the coefficient estimate, this statistic follows t distribution

Pr(>|t|): p-value for the t-test statistic

**Supplementary Table V:** Comparison of pathway features in the full-size (101 input pathways) and half-size (51 input pathways) pathway-based early-stage diagnosis models

Full-size pathway model:

| Features | Estimate | Std.Error | z value | Pr(>\|z\|) |
| --- | --- | --- | --- | --- |
| (Intercept) | -7.04E+01 | 2.54E+01 | -2.678 | 5.64E-03 |
| Taurine And Hypotaurine Metabolism* | 7.41 E+01 | 3.00E+01 | 2.469 | 1.36E-02 |
| Purine Metabolism* | 9.56E+01 | 4.22E+01 | 2.264 | 2.36E-02 |
| Propanoate Metabolism | 1.51E+01 | 7.20E+00 | 2.098 | 3.59E-02 |
| Protein Digestion And Absorption* | 4.46E+01 | 2.40E+01 | 1.856 | 6.35E-02 |
| CAMP Signaling Pathway | 4.25E+01 | 2.43E+01 | 1.754 | 7.95E-02 |
| Alanine Aspartate Glutamate Metabolism | -9.02E+01 | 5.63E+01 | -1.603 | 1.01E-01 |
| Biosynthesis Of Unsaturated Fatty Acids | 1.70E+01 | 1.13E+01 | 1.508 | 1.31E-01 |
| Malate-Aspartate Shuttle | -2.21E+01 | 1.99E+01 | -1.109 | 2.67E-01 |

Half-size pathway model:

| Features | Estimate Coefficient | Std.Error | z value | Pr(>\|z\|) |
| --- | --- | --- | --- | --- |
| Taurine And Hypotaurine Metabolism* | 2.86E+01 | 6.85E+00 | 4.177 | 2.95E-05 |
| (Intercept) | -1.23E+01 | 3.83E+00 | -3.214 | 1.31E-03 |
| Protein Digestion And Absorption * | 1.79E+01 | 9.62E+00 | 1.862 | 6.26E-02 |
| Histidine Metabolism | -1.75E+01 | 1.60E+01 | -1.094 | 2.74E-01 |
| Purine Metabolism* | 7.63E+00 | 1.15E+01 | 0.666 | 5.05E-01 |
| HIF-1 Signaling Pathway | 6.18E-01 | 3.46E+00 | 0.179 | 8.58E-01 |
| Beta-Alanine Metabolism | 1.78E+00 | 1.40E+01 | 0.127 | 8.98E-01 |

*: common pathways selected in both models

Estimate: Estimate coefficients

Std.Error: Standard error for estimated coefficients

Z-value: Wald statistic for the coefficient estimate, this statistic follows standard normal distribution

Pr(>|z|): p-value for the ward statistic
